# Breaking Solvation Dominance Effect Enabled by Ion–Dipole Interaction Toward Long-Spanlife Silicon Oxide Anodes in Lithium-Ion Batteries

**DOI:** 10.1007/s40820-024-01592-1

**Published:** 2024-12-26

**Authors:** Shengwei Dong, Lingfeng Shi, Shenglu Geng, Yanbin Ning, Cong Kang, Yan Zhang, Ziwei Liu, Jiaming Zhu, Zhuomin Qiang, Lin Zhou, Geping Yin, Dalong Li, Tiansheng Mu, Shuaifeng Lou

**Affiliations:** 1 State Key Laboratory of Space Power-Sources, School of Chemistry and Chemical Engineering, Harbin Institute of Technology, Harbin, 150001 People’s Republic of China; 2 School of Marine Science and Technology, Harbin Institute of Technology at Weihai, Weihai, 264200 People’s Republic of China; 3 Chongqing Research Institute of HIT, Chongqing, 401135 People’s Republic of China

**Keywords:** Lithium-ion batteries, Micrometer-sized silicon oxide, Ion-dipole interaction, Long-term cycling

## Abstract

Micrometer-sized silicon oxide (SiO) anodes encounter challenges in large-scale applications due to significant volume expansion during the alloy/de-alloy process. Herein, an innovative deep eutectic electrolyte derived from succinonitrile is introduced to enhance the cycling stability of SiO anodes. Density functional theory calculations validate a robust ion–dipole interaction between lithium ions (Li^+^) and succinonitrile (SN). The cosolvent fluoroethylene carbonate (FEC) optimizes the Li^+^ solvation structure in the SN-based electrolyte with its weakly solvating ability. Molecular dynamics simulations investigate the regulating mechanism of ion–dipole and cation–anion interaction. The unique Li^+^ solvation structure, enriched with FEC and TFSI^−^, facilitates the formation of an inorganic–organic composite solid electrolyte interphase on SiO anodes. Micro-CT further detects the inhibiting effect on the SiO volume expansion. As a result, the SiO|LiCoO_2_ full cells exhibit excellent electrochemical performance in deep eutectic-based electrolytes. This work presents an effective strategy for extending the cycle life of SiO anodes by designing a new SN-based deep eutectic electrolyte.

**Supplementary Information:**

The online version contains supplementary material available at 10.1007/s40820-024-01592-1.

## Highlights

- The succinonitrile-based deep eutectic electrolyte, characterized by strong ion–dipole interactions, can establish an anion-rich Li^+^ solvation structure while exhibiting high ionic conductivity and Li^+^ transference number.
- Precisely regulating multiple ion–ion, ion–dipole, and dipole–dipole interactions facilitates the transition of the Li^+^ solvation structure from solvent dominance to anion dominance.
- Optical microscopy and Micro-CT analysis can demonstrate that the anion-derived solid electrolyte interphase effectively mitigates the irreversible volume expansion of silicon oxide.

**Supplementary Information:**

The online version contains supplementary material available at 10.1007/s40820-024-01592-1.

## Graphical abstract

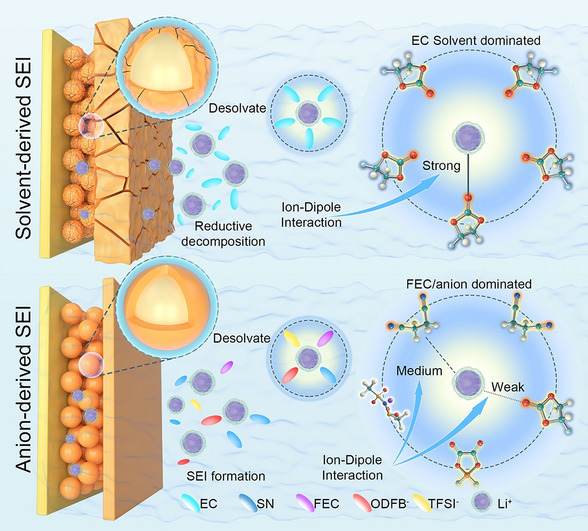

## Introduction

Given the escalating demand for energy concerns [1–3], developing rechargeable batteries with high-energy density and low cost is more and more imperative [4–6]. Silicon oxide (SiO) [7, 8] is highly suitable for commercial applications owing to its high capacity and processibility [9, 10]. Additionally, the SiO anodes exhibit decreased volume expansion during the charge and discharge cycles in compared to pure silicon [11, 12]. Despite the above advantages [13, 14], the SiO anodes still suffer from some limitations: (i) the low intrinsic electron conductivity; (ii) the irreversible formation of Li_2_O and lithium silicates; (iii) the residual volume expansion effect. In past years, many efforts primarily focused on mitigating volume expansion through material design [15], surface coating [16], and defect design [17]. Despite significant progress made in material modification studies, the solid–liquid interface compatibility in batteries remains inadequate, primarily attributed to the recurrent cracking tendencies of silicon-based anodes [18, 19]. Further investigation is still required to elucidate the interface reactions for constructing high-strength solid electrolyte interphase (SEI) between the SiO anodes and the electrolyte for long-spanlife lithium-ion batteries [20, 21].

The electrolyte, which plays a crucial role in silicon-based lithium-ion batteries, is essential for ensuring its long-term durability and maintaining SEI stability. In conventional liquid carbonate electrolytes (LE), the Li^+^ solvation sheath is primarily composed of organic solvent molecules such as ethylene carbonate (EC) and dimethyl carbonate (DMC) [6, 22, 23]. Typically, the decomposition of carbonate solvent molecules is preferentially initiated with a 1.0-M salt concentration in conventional carbonate solvents. This process leads to the formation of rich organic products with subpar mechanical properties, causing continuous electrolyte permeation and the accumulation of a thick SEI layer. The thick and unstable SEI film will aggravate the side reaction between electrolyte and electrode, resulting in further deterioration of electrochemical performance. Hence, optimizing the compatibility between the electrolyte and electrode through adjustments to the intrinsic Li^+^ solvation structure of the electrolyte holds paramount importance. Inspired by that, Chen group proposed the “drag effect” mechanism in solvents [24], where a self-adapting double-layer solvation structure increases the anion proportion within the Li^+^ solvation sheath. The introduction of low coordination number solvents into high coordination number solvent electrolytes can facilitate anions into the Li^+^ solvation sheath, creating the anion-derived SEI [25]. The anion-derived SEI has a high proportion of inorganic components, so it improves the mechanical strength of the SEI and restrains volume expansion of the SiO anode. Despite the considerable progress made in conventional electrolytes for silicon anodes, there is a pressing need to explore alternative solvents. Nitrile solvents demonstrate exceptional solubility with lithium salts, facilitating the development of a high ionic conductivity electrolyte system. Moreover, their remarkable high-voltage and thermal stability have the potential to improve cell safety [26, 27]. Understanding the interactions among cations, anions, and solvents, as well as the formation mechanism of the anion-rich Li^+^ solvation structure, is imperative for the establishment of a stable SEI layer on the silicon surface [28, 29].

In this study, we propose a mechanism for ion–dipole interaction in the novel deep eutectic electrolyte (ST electrolyte) composed of succinonitrile (SN) and LiTFSI for SiO anodes. In addition, we introduce fluoroethylene carbonate (FEC) solvent with weakly solvating ability (ST-F electrolyte) to modulate the ion–dipole interaction between Li^+^ and SN. The ST-F electrolyte exhibits notable contact ion pairs (CIP) and ion aggregates (AGGs) rather than isolated solvent-ion pairs (SSIP). In this electrolyte, the distinctive Li^+^ solvation structure, enriched with FEC and anions, preferentially decomposes on SiO anodes, resulting in the formation of a durable SEI rich in fluorine. This customized SEI plays a pivotal role in enhancing the interface stability and electrochemical performance utilizing SiO as an anode material [30]. The SEI effectively protects the anode from performance degradation caused by volume expansion and ongoing electrolyte decomposition [31]. Consequently, the SiO anodes demonstrate a significant capacity retention of 71.5% after 100 cycles in the ST-F electrolyte. Thus, the improved chemical stability and superior electrochemical performance of the ST-F electrolyte have the potential to propel the utilization of silicon-based anodes in high-energy–density batteries.

## Material and Methods

### Materials

#### Pristine Materials

Silicon oxide (SiO, btrchina), lithium cobaltate (LiCoO_2_, Canrud), succinonitrile (SN, 99%, Aladdin), carbonate electrolyte (1 M LiPF_6_ EC: DMC = 1:1 Vol%, dodo chem), fluoroethylene carbonate (FEC, 99%, Aladdin), lithium difluoro(oxalate)borate (LiDFOB, 99%, Aladdin), lithium bis(trifluoromethyl sulfonyl)imide (LiTFSI, 99%, Aladdin), N-methyl-2-pyrrolidone (NMP, 99.5%, Aladdin), polyvinylidene difluoride (PVDF) power, (HSV900, Arkema), PAA-Li (4%, Canrud)binder, and conductive carbon (Super C65).

#### Preparation of Electrolyte and Electrode

Deep eutectic electrolyte ST is synthesized by combining lithium salt and SN at a molar ratio of 1:15. After that, 0.75 mol of FEC is added to obtain the ST-F electrolyte. The SiO, PAA-Li binder, and Super P are mixed to form a homogeneous slurry with a weight ratio of 8:1:1. Subsequently, the slurry is poured onto a Cu foil current collector and subjected to vacuum oven drying at 80 °C for 12 h. The mass loading of SiO is about 1 mg cm^−2^; The LiCoO_2_ cathodes are prepared by combining LiCoO_2_, Super P, and PVDF binder at a mass ratio of 8:1:1 using NMP solvent. Subsequently, the slurry is coated onto the Al foil current collector and dried in a vacuum environment at a temperature of 120 °C for 12 h. The active substance has a bulk loading of approximately 3 mg cm^−2^.

### Methods

#### Assemblies and Measurements of Cells

The half cells are assembled using Celgard 2025 as the assembly material, with SiO or LiCoO_2_ employed as the working electrode, lithium foil utilized as the counter electrode, and glass fiber employed as the separator. The performance of the half cells is assessed using the NEWARE Battery Test System (CT-4008Tn-5V10mA-164, Shenzhen, China) at the temperature of 25 °C. The Li|SiO half-cell underwent cycling in the voltage range of 0.01 ~ 1.5 V, while the Li|LiCoO_2_ half-cell is cycled in the voltage range of 3.0 ~ 4.45 V (vs. Li^+^/Li).

The SiO anodes are initially pre-lithiated in the Li|SiO half-cell. After undergoing five cycles under a voltage range of 0.01 ~ 1.5 V at a current density of 0.1 A g^−1^, these half cells were discharged to 0.01 V. The pre-lithiated SiO anodes are then used as the anodes, and the complete cells are constructed by pairing high-pressure LiCoO_2_ (N/P = 1.15). The full cells, composed of SiO and LiCoO_2_, are cycled at 3 ~ 4.3 V.

#### Electrochemical Measurements

The cyclic voltammetry (CV) experiments use a voltage range of 0.01 ~ 1.5 V and a scanning rate of 0.1 mV s^−1^. The linear scanning voltammetry (LSV) is conducted using a voltage range of 3 ~ 6 V and a scanning rate of 1 mV s^−1^. The ionic conductivity ($\sigma$) of the electrolyte is determined by conducting electrochemical impedance spectroscopy (EIS) measurements. An alternating current (AC) voltage of 5 mV is applied throughout a frequency range of 0.1 to 10^5^ Hz. The electrolyte is placed between two stainless-steel (SS) platelets. The $\sigma$ is determined by applying the equation in which L represents the thickness of the electrolyte membrane, $R$ denotes the resistance measured by EIS, and $S$ means the contact area between the electrolyte membrane and SS.

1 $$\sigma = \frac{d}{{\left( {R \cdot S} \right)}}$$

The Li^+^ transference number of the electrolyte is determined by combining chronoamperometry and EIS measurements of the Li|Li cell. The value is computed using the following mathematical equation: The polarization voltage (10 mV) utilized in this study is denoted as $V$. ${I}_{0}$ and ${I}_{s}$ denote the initial and steady-state current, correspondingly ${R}_{0}$ and ${R}_{s}$ denote the initial and equilibrium impedance, respectively.

2 $$t = \frac{{I_{s} \left( {V - I_{0} R_{0} } \right)}}{{I_{0} \left( {V - I_{s} R_{s} } \right)}}$$

#### Material Characterizations

The morphology and structure of cycled SiO anodes are analyzed via various advanced techniques, including scanning electron microscope (SEM, Hitachi S4700), transmission electron microscope (TEM, FEI/Philips TCNAI G2), X-ray photoelectron spectroscopy (XPS, PEI Quantum 2000), thermogravimetric analyzer (TG, F3 Jupiter), Fourier transform infrared spectrometer (FT-IR, NICOIIS10), Raman (Renishaw Syem 1000), and Micro-CT (BL13W1 beamline of SSRF at 15 keV).

#### Theoretical Calculation

The density functional theory (DFT) computations are executed using Gaussian 09 [36]. The geometry optimizations are performed using a hybrid B3LYP-D3(BJ) density functional [37] and a 6-31G basis set [38], resulting in precise geometries. The molecular structure of various electrolyte components is initially optimized until the forces reach a value of 4.5 × 10^–4^ Hartrees/Bohr^−1^. The more accurate M06-2X-D3 [39]/6-311G [40, 41] level is employed to calculate single-point energies, utilizing ideal molecular configurations. The binding energies are computed using the equation, in which ${E}_{complete}$ represents the overall energy of the complex and ${E}_{frag}$ denotes the energy of each fragment. The VMD [42] program is utilized to visualize data.

3 $$E_{b} = E_{complete} - E_{frag}$$

Molecule dynamics (MD) simulations are conducted using Gromacs (version 2023.5). For the ST electrolyte components, 1500 SN and 100 LiTFSI molecules are added to an 8 × 8 × 8 nm^3^ simulation box, following the experimental density and stoichiometry. Similarly, for the ST-F electrolyte, 75 FEC, 1500 SN, and 100 LiTFSI molecules are added to an 8 × 8 × 8 nm^3^ simulation box, following the experimental density and stoichiometry. The OPLS-AA force field is used in this study to describe the inter- and intra-molecular interactions. The parameters for all molecules are automatically generated using the LigParGen Server. All force field parameters are provided in.itp files, along with the nonbonding parameters for solvent molecules. All simulations begin with a 2 ns NPT run at 500 K, followed by a 3 ns NPT annealing process, during which the system temperature decreases from 330 K to room temperature (298 K). This process results in the preparation of a homogeneous single-phase solution as the initial configuration. Subsequently, the system is equilibrated through a 5 ns NPT simulation and a 10 ns NVT simulation, followed by a 5 ns production run for RDF. The temperature and pressure are controlled using the Nose–Hoover thermostat and Berendsen barostat, respectively. Additionally, a time step of 1.0 fs is employed in all simulations. The MD trajectories are visualized using VMD to obtain snapshots of the system at various time points [34].

## Results and Discussion

### Solvation Structure and Chemistry of the SN-Based Electrolytes

**Fig. 1:**
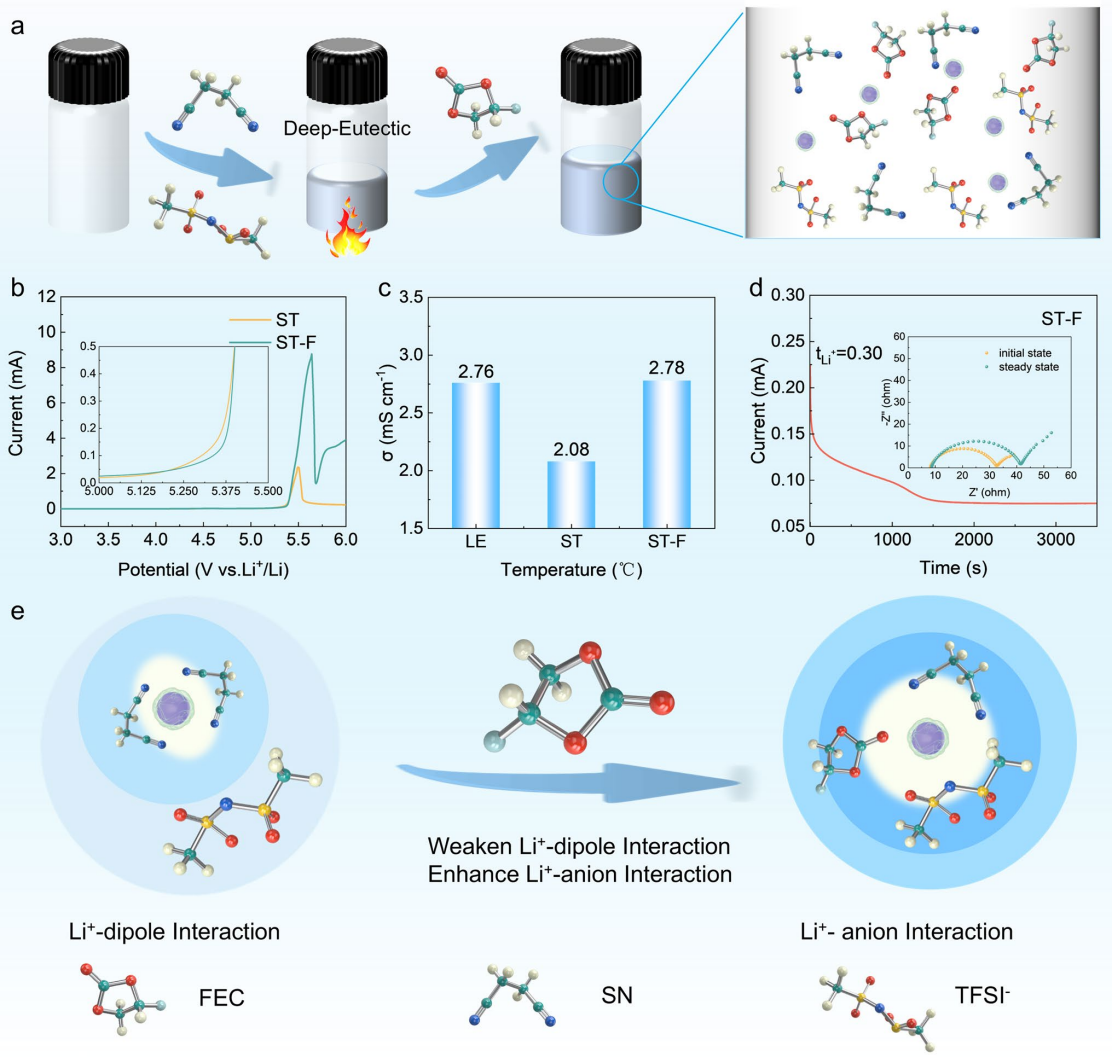
Preparation and electrochemical properties of SN-based electrolyte. **a** Schematic illustration of the formation principle of SN-based deep eutectic electrolyte. Electrochemical property tests: **b** LSV curve at 0.5 mV s^−1^. **c** Ionic conductivity at room temperature. **d** Li^+^ transference number test. **e** Schematic illustration of the action of FEC

Figures 1a and S1 show a schematic illustration of the formation principle of the liquid SN-based deep eutectic electrolyte, where LiTFSI and SN are solid state at ambient temperature [32]. In the ST electrolyte (SN:LiTFSI = 15:1 by mol), SN solvent molecules enter the Li^+^ solvation sheath layer and then form a stable electrolyte system due to the polarity and electronegativity derived from the C≡N bond [33]. Benefiting from the good electrochemical stability of SN, the electrochemical windows of SN-based electrolytes are extended to 5.0 V and exceed that of LE electrolytes (Figs. 1b and S2). This distinction arises from the increased stability of the ion–dipole interaction between succinonitrile and lithium salts. The ST electrolyte and ST-F electrolyte (SN/LiTFSI/FEC = 15:1:0.75 by mol) exhibit room-temperature conductivity of 2.08 and 2.78 mS cm^−1^ (Fig. 1c) along with Li^+^ transference number of 0.21 and 0.30 (Figs. 1d and S3). Within the Li^+^ solvation sheath layer, various interactions exist as competitive relationships, encompassing ion–ion, ion–dipole, and dipole–dipole interactions [34, 35]. The strong Li^+^–dipole interaction in the ST and LE electrolytes leads to a reduced concentration of TFSI^−^ attributed to the weak cation–anion interaction within the Li^+^ primary solvation sheath layer. The incorporation of FEC results in a diminished ion–dipole interaction with Li^+^, facilitating enhanced interaction between Li^+^ and TFSI^−^, thereby causing a substantial influx of free TFSI^−^ into the primary solvation sheath layer to create a distinctive Li^+^ solvating structure enriched in anions (Fig. 1e).

**Fig. 2:**
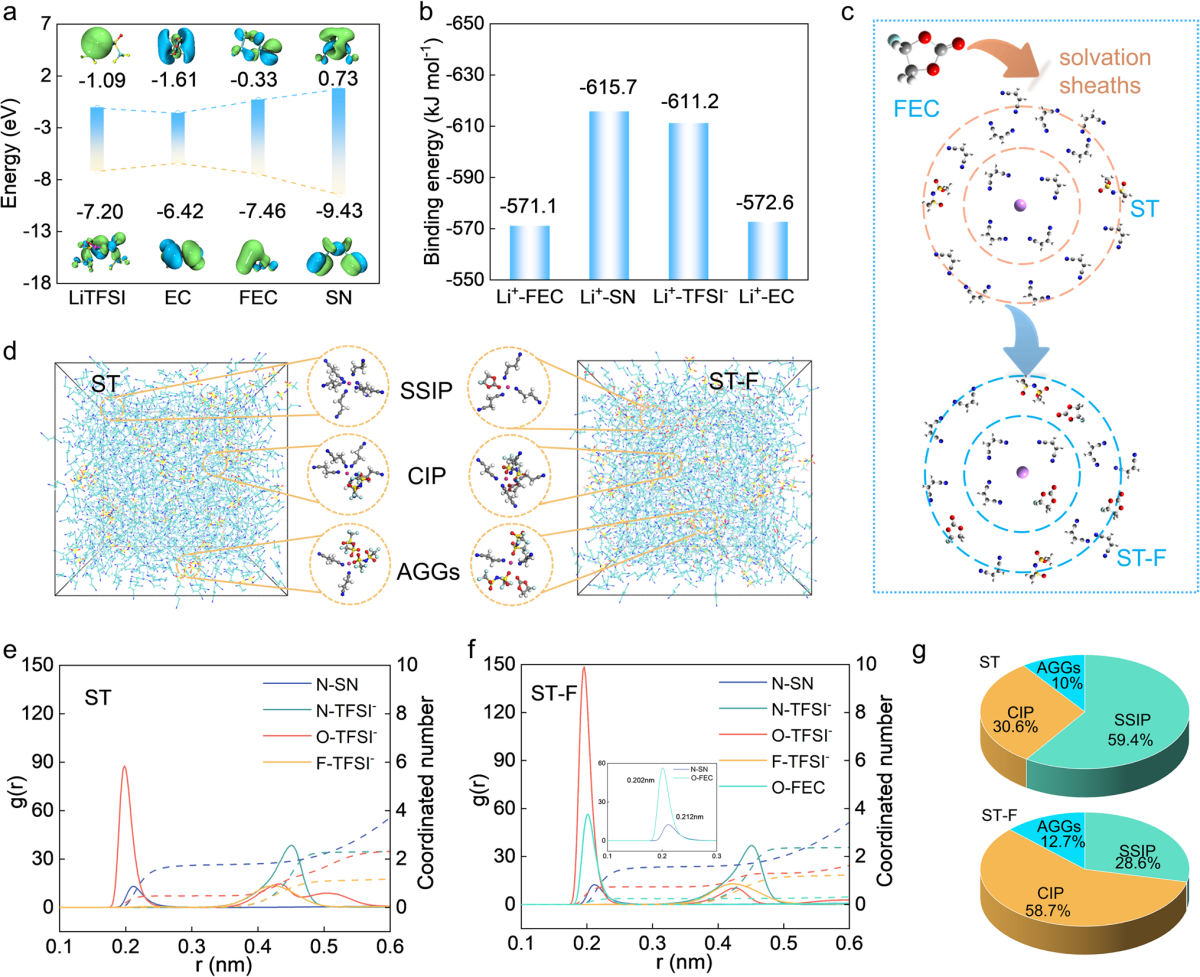
DFT calculation and solvation structure analysis of SN-based electrolyte. **a** LUMO and HOMO energy levels. **b** Binding energies of Li^+^. **c** Schematic illustration of FEC regulates SN-based electrolyte the Li^+^ solvation structure. **d** MD simulation snapshots of ST and ST-F electrolyte. RDF of different electrolytes: **e** ST electrolyte. **f** ST-F electrolyte. **g** Comparison of ratios of SN-based electrolytes SSIP, CIP, and AGGs

DFT calculation is first carried out to investigate the solvation structure mode of the SN-based electrolyte. From the electrostatic potential distribution (ESP), the nitrogen atoms in SN and the oxygen atoms in FEC demonstrate a significant tendency to form coordination with Li^+^ due to their relatively high electron cloud densities Fig. S4. Figure 2a illustrates the computed LUMO and HOMO energy levels of the SN solvent and additional electrolyte constituents. The comparatively lower LUMO energy levels of EC (-1.61 eV), LiTFSI (-1.09 eV), and FEC (-0.33 eV) suggest a heightened reduction likelihood in contrast to SN (0.73 eV). Furthermore, the lower HOMO energy levels of SN (-9.43 eV) compared to other electrolyte components signify robust antioxidant characteristics and significant potential for high-voltage batteries. From Fig. 2b, the binding energy of Li^+^-FEC is lower than those of Li^+^-EC and Li^+^-SN, indicating a weakly solvating ability of FEC. It is known that the Li^+^ solvation structure directly influences the stability of the electrolyte/electrode interface. The solvents and anions in the Li^+^ solvation sheath are more likely to approach the electrode than free solvents and anions, determining the main components of the formatted SEI. Figure 2c depicts the role of FEC within the Li^+^ first solvation sheath layer of the ST-F electrolyte, where the FEC reduces the ion–dipole interaction between Li^+^ and SN solvent molecules while moderately enhancing the interaction between Li^+^ and anions (TFSI^−^).

Further, the Li^+^ solvation structure in ST and ST-F electrolytes is analyzed through molecular dynamics simulations (MD) in Fig. 2d, where typical Li^+^ solvation structures, including Li^+^(SN)_5_, Li^+^(SN)_4_(TFSI^−^), and Li^+^(SN)_2_(TFSI^−^)_2_, are identified in the ST electrolyte. Distinct solvation structures are generated upon the addition of FEC to the ST electrolyte, including Li^+^(SN)_3_(FEC), Li^+^(SN)_3_(TFSI^−^), and Li^+^(SN)_2_(TFSI^−^)_2_(FEC). In the SN-based electrolyte, the Li^+^ first solvation sheath is approximately 0.25 nm, and the second solvation sheath is about 0.5 nm **(**Fig. 2e, f**)**. In the ST electrolyte, SN predominantly occupies the first solvation sheath with a coordination number of 1.61 for Li^+^-N(SN), while TFSI^−^ is only a coordination number of 0.48 for Li^+^-O(TFSI^−^). In the ST-F electrolyte, the initial Li^+^-N(SN) peak appears around 0.202 nm, while the initial Li^+^-O(FEC) peak is around 0.212 nm. The above results indicate that FEC is more likely to access the Li^+^ first solvation sheath layer than SN by approximately 0.25 nm. Importantly, the Li^+^-O(TFSI^−^) coordination number markedly increases to 0.74, indicating that FEC weakens the ion–dipole (Li^+^-N) interaction in the Li^+^ solvation sheath and enhances the ion–ion interaction (Li^+^-TFSI^−^). It is concluded that due to the weaker ion–dipole interaction of Li^+^-FEC compared to Li^+^-SN, the Li^+^-TFSI^−^ interaction is enhanced in the ST-F electrolyte, leading to a more substantial involvement of anions in the Li^+^ solvation structure. Furthermore, the proportion of SSIP, CIP, and AGGs with varying coordination states near Li^+^ is quantified in Fig. 2g. The proportion of AGGs and CIP in the ST-F electrolyte is 12.7% and 58.7%, higher than that in the ST electrolyte (10% and 30.6%). Therefore, we can conclude that a positive effect on weakly solvating solvent FEC effectively promotes the formation of anion-derived solvation structure.

**Fig. 3:**
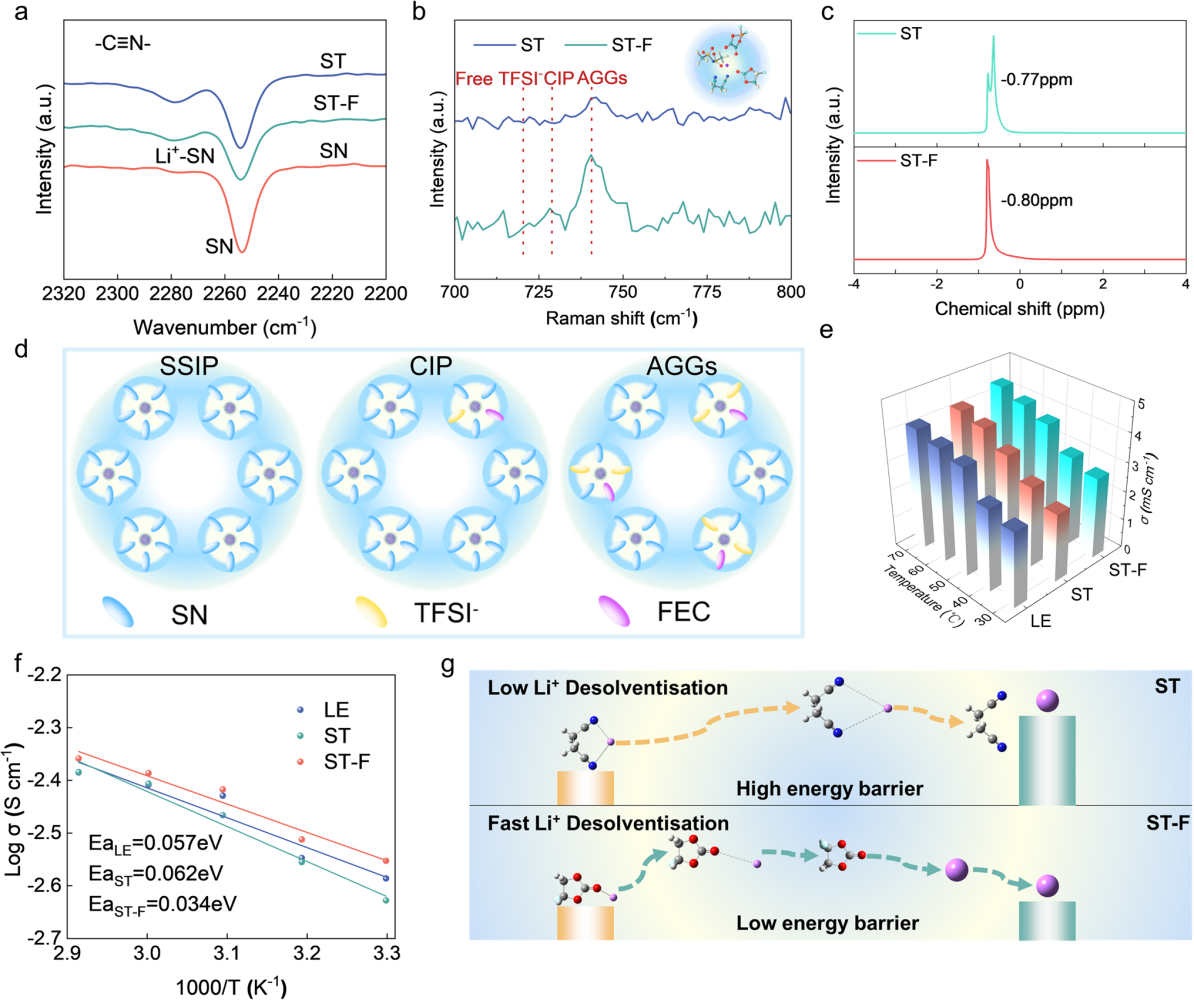
Li^+^ solvation structures and migration kinetic analysis. **a** Fourier variation Infrared spectroscopy test. **b** Raman spectra test. **c** ^7^Li NMR spectra test. **d** Schematic illustration of SSIP, CIP, and AGGs. **e** Ion conductivity test at different temperatures. **f** Arrhenius plot for the resistance of Li^+^ migration. **g** Schematic illustration of the desolvation of Li^+^ in ST and ST-F electrolyte

Furthermore, Fourier transform infrared spectroscopy and Raman are employed to analyze the distinct Li^+^ solvation structures in the SN-based electrolyte (Fig. S5). The infrared spectrum presents two typical peaks at approximately 2950 and 2253 cm^−1^, corresponding to the stretching vibration of C-H and the stretching vibration of C≡N in SN (Fig. 3a). The C≡N peak split at 2280 cm^−1^ due to the interaction of Li^+^ to SN in the ST and ST-F electrolytes (Fig. 3b). In the Raman spectrum, characteristic peaks at 721, 729, and 741 cm^−1^ are assigned to free TFSI^−^, CIP, and AGGs. A higher proportion of the AGGs is observed in the ST-F electrolyte compared to the ST electrolyte. Then, ^7^Li liquid NMR spectrum is carried out to deeply elucidate the Li^+^ solvation environment (Fig. 3c). The introduction of FEC induced a downshift in the resonance signal to -0.80 ppm, indicating an increased anion concentration around Li^+^. Figure 3d illustrates the typical solvation structure of SSIP, CIP, and AGGs, indicating that in AGGs and CIP, a higher number of anions are present in the first solvation sheath layer compared to SSIPs. Figures 3e and S6 show the diffusion kinetics of Li^+^ in the different SN-based electrolytes are through EIS at various temperatures. The Li^+^ diffusion kinetics in the LE electrolyte is sluggish due to the robust ion–dipole interaction and the high-energy barrier for Li^+^ desolvation. The ST-F electrolyte exhibits a lower diffusion barrier (0.034 eV) and superior Li^+^ diffusion kinetics than the LE electrolyte and ST electrolyte (Fig. 3f). Figure 3g illustrates the Li^+^ desolvation process within the SN-based electrolyte. The robust ion–dipole interaction between SN and Li^+^ elevates the migration barrier for Li^+^. Conversely, FEC demonstrates superior Li^+^ diffusion kinetics attributed to its weak solvating capability and reduced desolvation barrier.

### Interface Variations and Cell Performances

**Fig. 4:**
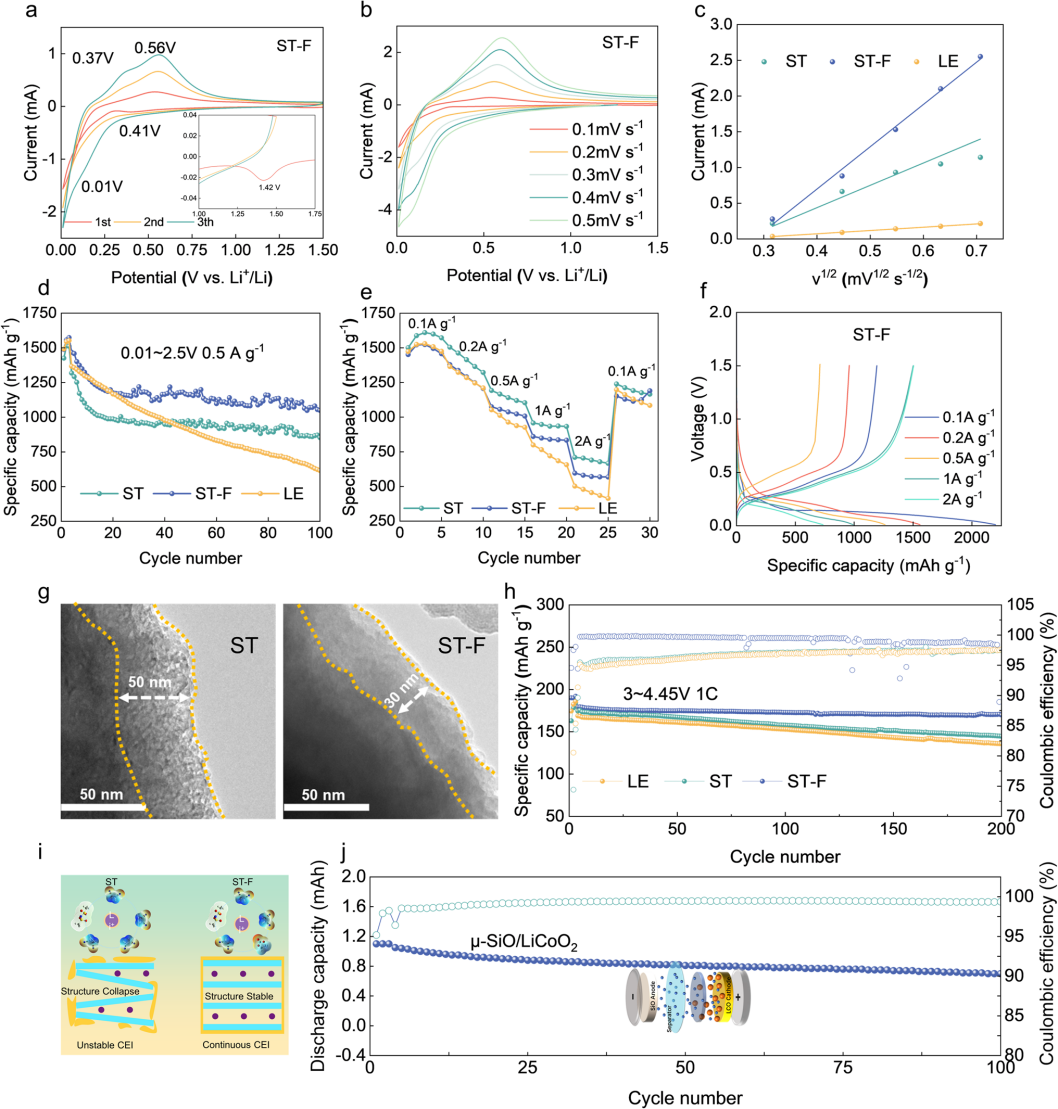
Electrochemical performance of the Li|SiO, Li|LiCoO_2_ and SiO|LiCoO_2_ cells. **a** The CV curves of ST-F electrolyte. **b** CV curves of SiO anode in ST-F electrolyte with different sweep speeds. **c** The linear relationship between peak current and sweep speed square root. **d** Cycling performance of Li|SiO batteries at 0.5 A g^−1^. **e** Rate performance of Li|SiO cells ranging from 0.1 A g^−1^ to 2 A g^−1^. **f** Charge and discharge curve of SiO anode in ST-F electrolyte at 0.1 to 2 A g^−1^. **g** TEM images of the cycled SiO anodes with SN-based electrolyte. **h** Cycling performance of Li|LiCoO_2_ batteries at 1 C. **i** Schematic illustration of the ST/ST-F electrolyte action mechanism on the LiCoO_2_. **j** Cycling performance of SiO|LiCoO_2_ batteries at 0.5 C

The cyclic voltammetry tests of the SiO anode are performed in half-cell (SiO/Li) configurations in the corresponding electrolytes to reveal the alloy/de-alloy behavior of the SiO anodes. In the ST electrolyte, a peak at 1.38 V during the initial alloy can be attributed to the LiTFSI decomposition (Fig. S7). The broad reduction peak near 0.5 V is concluded to the SEI formation during the initial lithium insertion process. The typical reduction peak at 0.01 V can be attributed to the formation of Li_2_O and Li_x_Si. The decomposition peak at 1.42 V in the ST-F electrolyte could be assigned to the synergistic decomposition effect of LiTFSI and FEC (Fig. 4a). It is suggested that SiO anodes exhibit typical alloy/de-alloy behavior in the SN-based electrolytes. A distinct reduction peak emerged at approximately 1.21 V in the LE electrolyte, which can be attributed to the reduction of EC decomposition. The alloying/de-alloying kinetics of SiO anodes in the different SN-based electrolytes are examined through cyclic voltammetry curves at various sweeps (Figs. 4b and S8). The SiO anodes in the ST-F and ST-F electrolyte exhibit excellent alloy/de-alloy kinetics (Fig. 4c), primarily owing to the decreased Li^+^ desolvation energy and migration resistance.

In Fig. 4d, the SiO anodes exhibit initial discharge capacities of 1425.7, 1488.4, and 1492.3 mAh g^−1^ in the ST, ST-F, and LE electrolyte. The electrochemical impedance spectrum reveals a relatively stable state of SiO in the ST-F electrolyte (Fig. S9). This stability can be ascribed to the synergistic reduction and decomposition of the rich-FEC/anion solvation structure to form a robust and compact SEI. The microscopic morphology of the cycled SiO anodes is further analyzed through SEM (Fig. S10). Numerous micropores displayed on the SiO anodes in the LE electrolyte indicate a significant side reaction between the EC and the SiO anodes. Meanwhile, a significant capacity decrease in the ST electrolytes can be attributed to the inadequate protection offered by the solvation-derived SEI against substantial volume expansion, which is also observed in the LE electrolytes. In contrast, the SiO anodes cycled in the ST-F electrolyte exhibit uniform surface morphology without visible fractures and obvious volume expansion. In Figs. 4e and S11, it is evident that the SiO anodes demonstrate a favorable rate performance in the ST-F electrolyte, maintaining a specific capacity of 822.1 mAh g^−1^ at a current density of 2 A g^−1^. In contrast, the ST electrolyte displayed a residual capacity of 596.1 mAh g^−1^, and the LE electrolyte demonstrated a residual capacity of 502.2 mAh g^−1^ (Fig. 4f).

The Si/C anodes also demonstrate excellent cycling performance in the ST-F electrolyte, with minimal capacity decay even after 100 cycles at a current density of 0.5 A g^−1^ (Fig. S12). In Fig. 4g. The SiO anodes exhibit severe side reactions, leading to a thick SEI of about 50 nm in the ST electrolyte. In contrast, the anion-derived solvating structure in the ST-F electrolyte promotes a compact SEI formation with a thickness of 30 nm. The SN-based electrolytes demonstrate outstanding thermodynamic stability with a mass loss of less than 5% before reaching 174 °C. The SN-based electrolyte tolerates significant degradation primarily at 200 °C, suggesting that the electrolyte can maintain stability even at high temperatures. Moreover, the SiO anodes exhibit good electrochemical performance at high temperatures in the ST-F electrolytes (Fig. S13). For the LiCoO_2_, it also demonstrates excellent high-voltage performance with 95.2% capacity retention after 150 cycles in the ST-F electrolyte, due to a stable complexation between C≡N and the transition metal ions ensuring the consistent cycling of the LiCoO_2_ cathode (Fig. 4h, i). Consequently, the assembled SiO|LiCoO_2_ full cells with the ST-F electrolyte demonstrate excellent cycling stability. This implies that the SEI/CEI, formed by adjusting the ion–dipole interaction to create an anion-rich solvated structure, showcases strong stability, consequently prolonging the battery's lifespan (Fig. 4j).

**Fig. 5:**
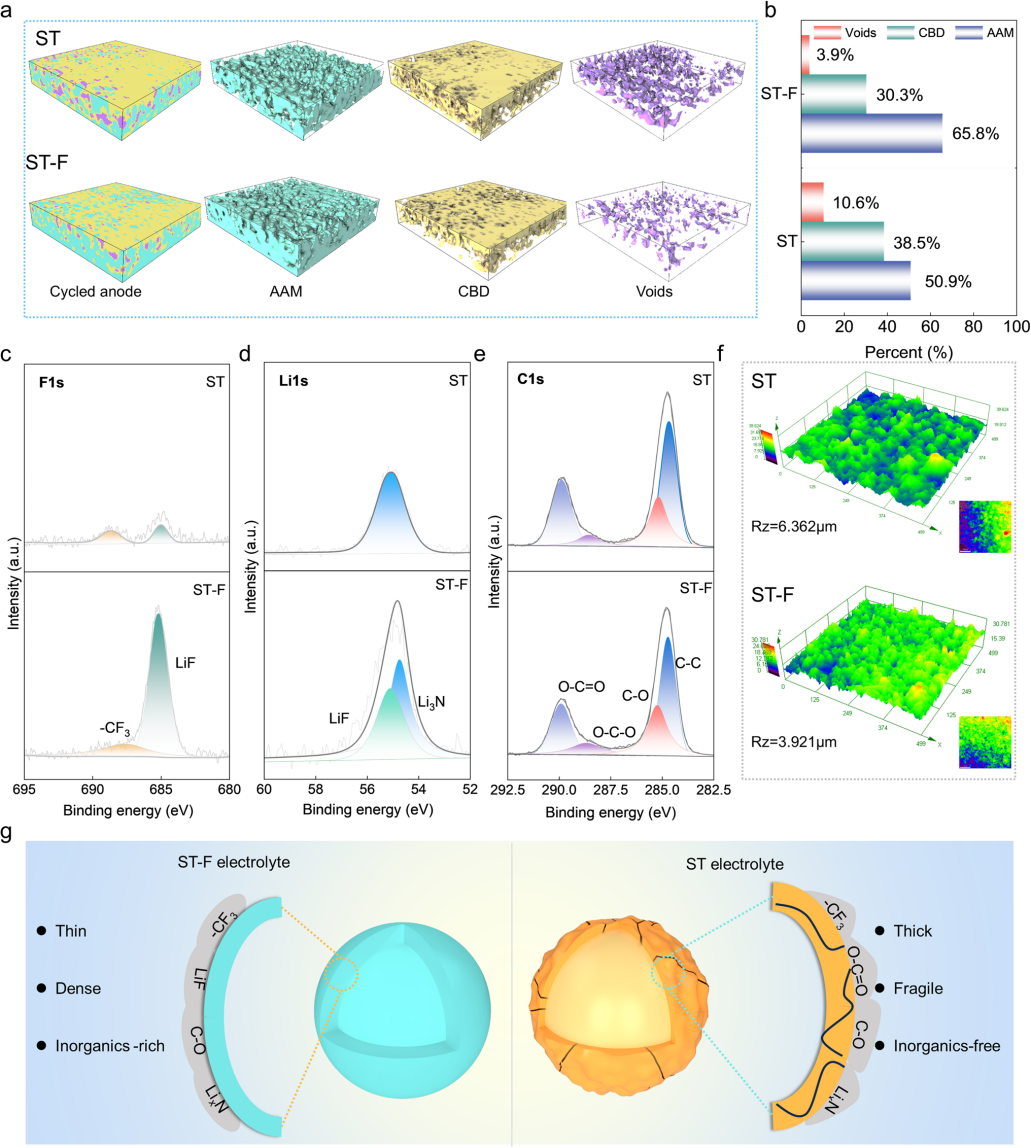
Components structure and surface chemistry of cycled SiO anodes. **a** Synchrotron X-ray computed tomography reconstruction with volume rendering shows the 3D images and volume fraction of SiO anodes after cycling in SN-based electrolyte. **b** Percentage of each component, XPS spectra of SiO anodes after cycling in the SN-based electrolyte. **c** Li 1*s*. **d** F 1*s*. **e** C 1*s*. **f** Roughness test of SiO anodes after cycling in the ST/ST-F electrolyte. **g** Schematic illustration of the SEI component of the SiO anodes surface in the SN-based electrolyte

Synchrotron X-ray computed tomography is used to analyze and quantify the distribution of different components in the cycled SiO. The 3D microstructure of the SiO anodes is constructed using X-ray attenuation data obtained from multiple regions (Fig. 5a). The images of the cycled SiO anodes are divided into sections with dimensions of 100 × 20 × 100 μm^3^. These segments illustrate the distribution and proportion of three components consisting of the SiO network (high attenuation), binder & conductive carbon network (low attenuation), and voids (zero attenuation). The SiO network, binder, and conductive carbon network collectively form a rapid percolation network for electronic and ionic conduction. The voids resulting from volume expansion during the cycling process hinder the movement of ions and electrons, leading to sluggish ion transport and uneven electrode response kinetics. In Fig. 5b, the voids of the cycled SiO anodes in the ST-F electrolyte (3.9 vol%) are smaller than those in the ST electrolyte (10.6 vol%). The SEM images also verify that the SEI formed in the ST-F electrolyte can effectively mitigate the volume expansion. The SEI components of the SiO cycled in the SN-based electrolyte are analyzed by XPS. The F 1*s* spectra of cycled SiO in the ST electrolyte exhibit a weak -CF_3_ signal (688.7 eV) (Fig. 5c) attributed to the TFSI^−^ dissolution. The fluorine element reacts with Li and produces in a LiF signal (684.9 eV) in the ST-F electrolyte. The analysis of the Li 1*s* spectra also displays the signals of Li_x_N (54.7 eV) and LiF (55.1 eV), indicating the formation of rich in FEC and anion-derived SEI on the SiO anodes in the ST-F electrolyte. This results in the generation of a greater quantity of LiF, consequently enhancing SEI stability. FEC, rich in fluorine content, forms a durable and adaptable organic–inorganic composite SEI, thereby boosting electrochemical performance. The C 1*s* spectrum also shows various carbon-containing components signals (Fig. 5e). Optical microscopy experiments reveal that the cycled SiO electrodes in the ST-F electrolyte exhibit a flatter and smoother morphology (R_Z_ = 3.921) compared to the ST electrolyte (R_Z_ = 5.362), which can be attributed to the formation of a denser and more uniform anion-derived SEI (Fig. 5f). In the ST electrolyte, the electrolyte undergoes continuously reductive decomposition on the SiO anodes, resulting the SEI primarily composed of -CF_3_, O-C = O, C-O, Li_x_N, and other substances (Fig. 5g). Moreover, the surface of the SiO anodes cycled in the ST-F electrolyte exhibits more organic–inorganic components, including -CF_3_, LiF, and Li_x_N, which can efficiently inhibit the constant volume expansion and the fragmentation.

**Fig. 6:**
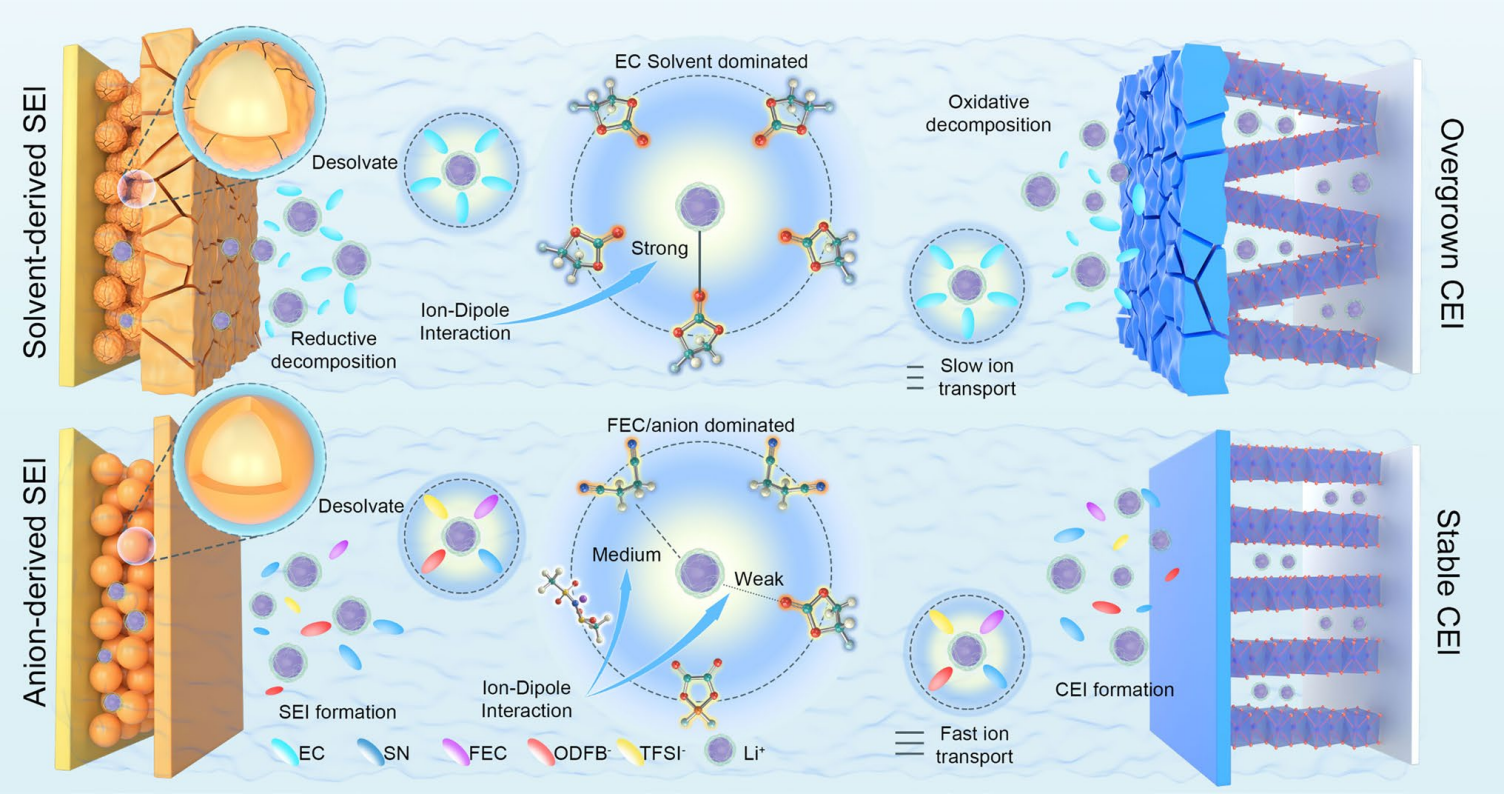
Schematic illustration of the mechanism EC/SN electrolyte at the electrode interfaces

Figure 6 illustrates typical Li^+^ solvating structures and the film-formed mechanism of the cells in the LE and SN-based electrolytes. It is suggested that in the LE electrolyte, Li^+^ solvation structures predominantly governed by the solvent lead to EC continuous reduction and decomposition on the electrode surface, causing an unstable SEI and safety risks. Moreover, the unstable cathode electrolyte interface (CEI) is susceptible to recurrent rupture, remodeling, oxidation, and decomposition in the LE electrolyte. The strong ion–dipole interaction between Li^+^ and EC/SN molecules in the LE/ST electrolyte facilitates lithium salt dissociation but restricts Li^+^ migration. By introducing of FEC to regulate the ion–dipole interaction between SN and Li^+^, the Li^+^ solvation structure enriched with FEC and anions is established. The solvation structure promotes the formation of anion-derived inorganic-rich SEI/CEI on electrodes. The rigid LiF layer effectively suppresses the volume expansion of the SiO anodes and oxygen precipitation from the LiCoO_2_ lattice during the cycling process.

## Conclusion

In summary, we report a new nitrile-based electrolyte derived from SN for an anion-dominated solvating structure, aiming to enhance the long-term cycling performance of 4.45 V SiO**|**LiCoO_2_ cells. DFT calculations and MD simulations have been performed to modulate microscopic forces (ion–ion, ion–dipole, and dipole–dipole interactions) within the SN-based electrolyte, enabling the construction of anion-rich solvation structures even with lithium salts. The Li^+^ solvation structure is precisely regulated at the molecule level in the SN-based electrolyte by introducing weakly solvating solvent FEC, realizing higher conductivity and mobility than the LE electrolyte. That addresses the problem imposed by the solvent-dominated solvation structure prevalent in LE electrolytes, thereby facilitating the application of silicon-based materials into SN-based electrolytes. Micro-CT demonstrates that anion-rich derived SEI could effectively inhibit the irreversible volume expansion of SiO during the alloying/de-alloying process. These findings robustly endorse the capability of SN-based electrolytes to facilitate the practical deployment of high-capacity and silicon-based lithium-ion batteries.

## Supplementary Information

Below is the link to the electronic supplementary material.

Supplementary file1 (DOCX 3557 KB)
