# Supplementary material for: Breaking Solvation Dominance Effect Enabled by Ion–Dipole Interaction Toward Long-Spanlife Silicon Oxide Anodes in Lithium-Ion Batteries
Source: Nanomicro Lett. 2024 Dec 26;17:95. doi: 10.1007/s40820-024-01592-1 (PMC11671450; doi:10.1007/s40820-024-01592-1)
Supplement: Supplementary file 1 — Supplementary file1 (DOCX 3557 KB) [file 40820_2024_1592_MOESM1_ESM.docx]

Supporting Information for

**Breaking Solvation Dominance Effect Enabled by Ion-Dipole Interaction towards Long-Spanlife Silicon Oxide Anodes in Lithium-Ion Batteries**

Shengwei Dong^1^, Lingfeng Shi^1^, Shenglu Geng^1^, Yanbin Ning^1^, Cong Kang^1^,Yan Zhang^1^, Ziwei Liu^1^, Jiaming Zhu^1^, Zhuomin Qiang^1^, Lin Zhou^2^, Geping Yin^1^, Dalong Li^2,^ *, Tiansheng Mu^1,^ *, Shuaifeng Lou^1, 3,^*

^1^ State Key Laboratory of Space Power-Sources, School of Chemistry and Chemical Engineering, Harbin Institute of Technology, Harbin 150001, P. R. China

^2^ School of Marine Science and Technology, Harbin Institute of Technology at Weihai, Weihai 264200, P. R. China

^3^ Chongqing Research Institute of HIT, Chongqing 401135, P. R. China

*Corresponding authors. E-mail: [lidalong@hit.edu.cn](mailto:lidalong@hit.edu.cn) (Dalong Li); [mutiansheng@hit.edu.cn (Tiansheng Mu); shuaifeng.lou@hit.edu.cn](mailto:mutiansheng@hit.edu.cn%20(Tiansheng%20Mu);%20shuaifeng.lou@hit.edu.cn) (Shuaifeng Lou)

**Supplementary Figures**


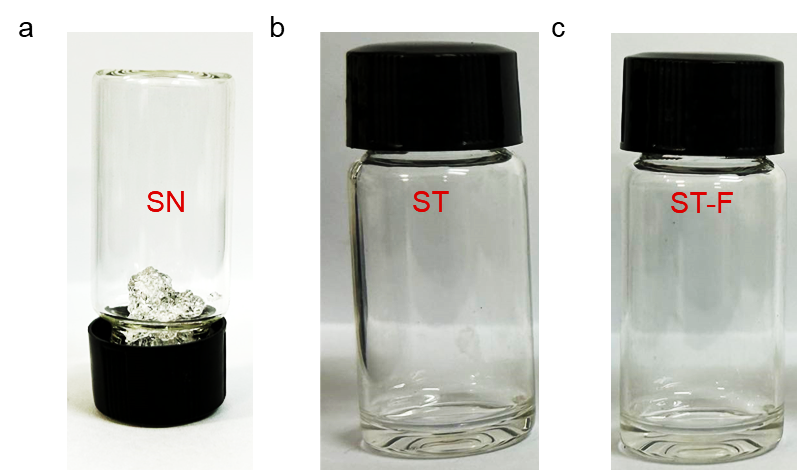


**Fig. S1** Digital photos of SN and SN-based electrolytes. (**a**) SN, (**b**) ST, (**c**) ST-F


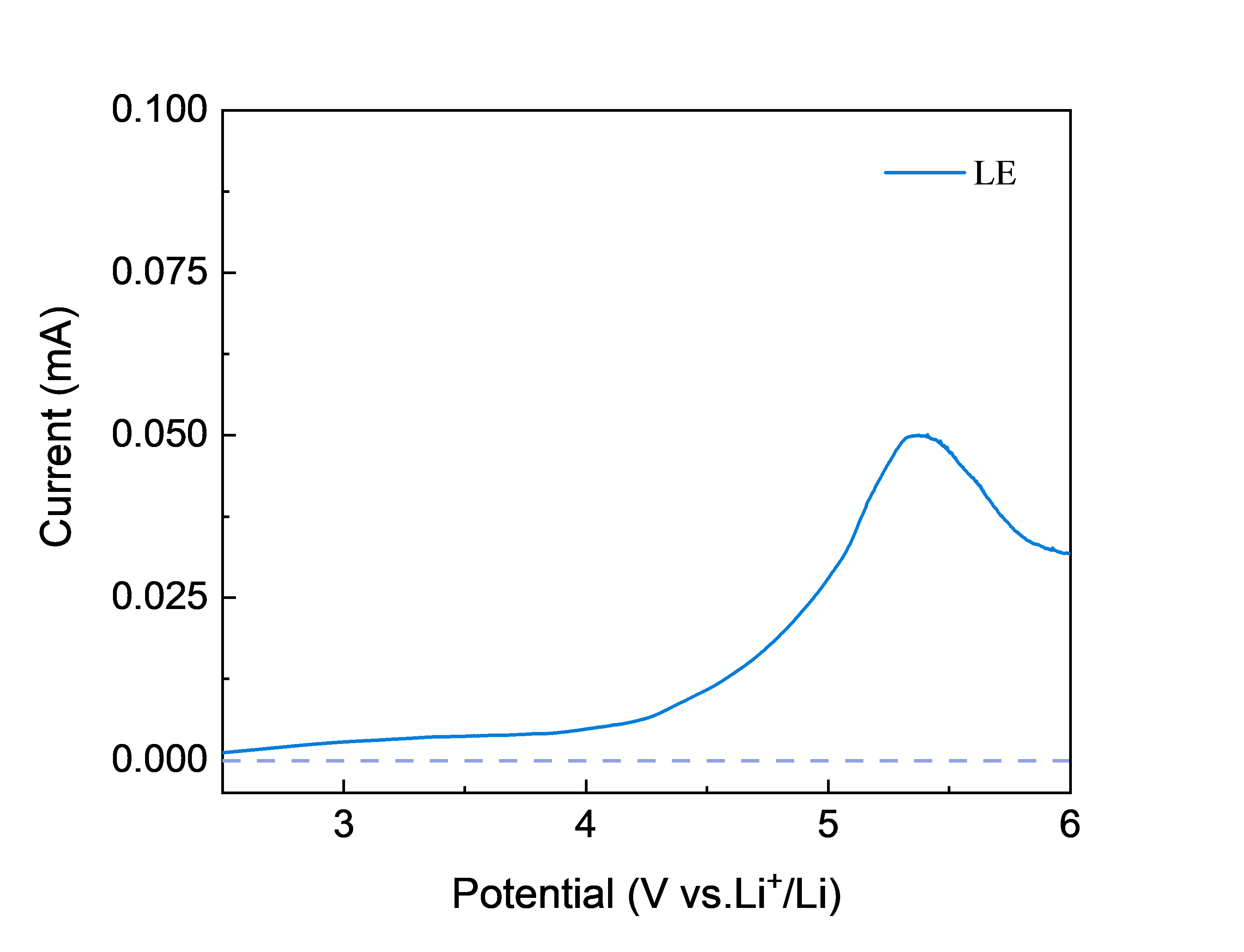


**Fig. S2** LSV curves of LE


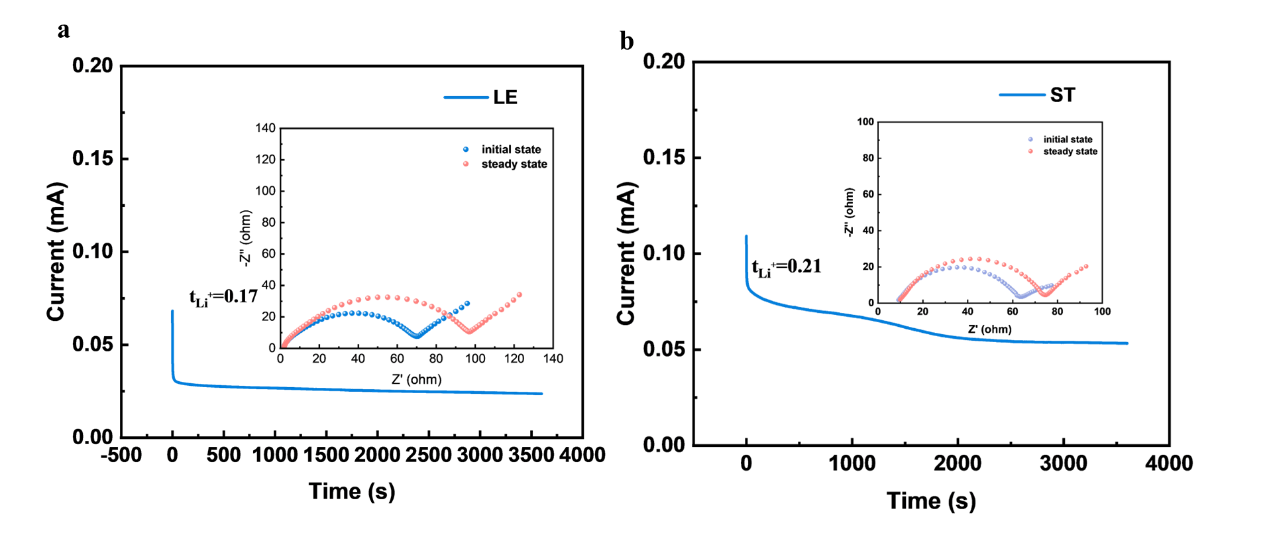


**Fig. S3** Transference number of the electrolytes. (**a**) LE, (**b**) ST


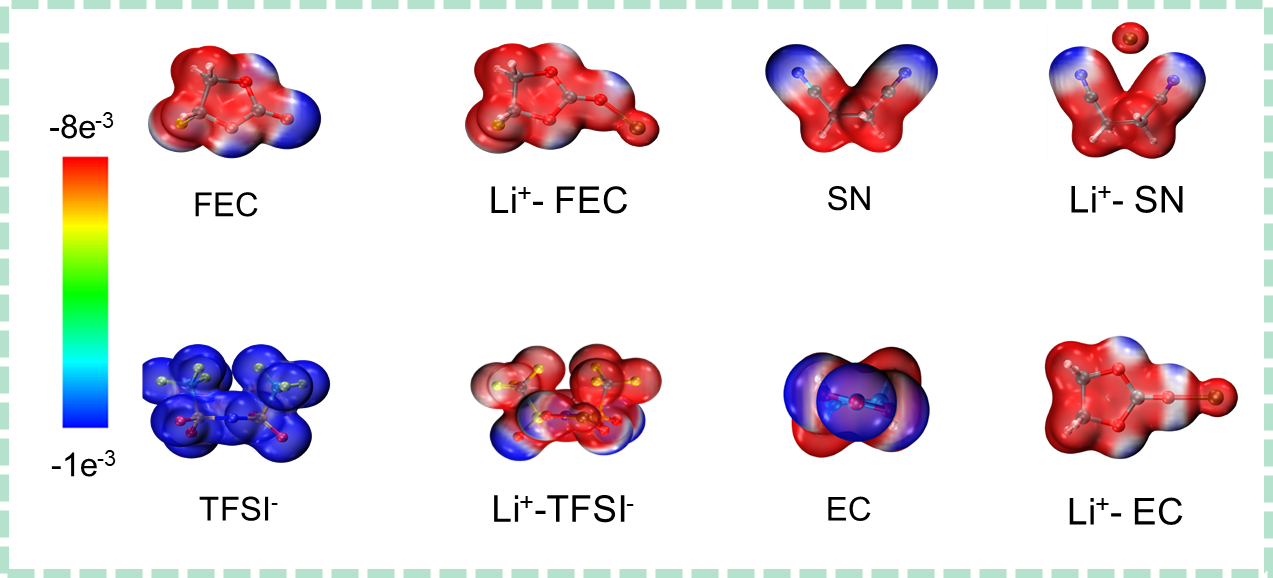


**Fig. S4** Electrostatic potential density distribution


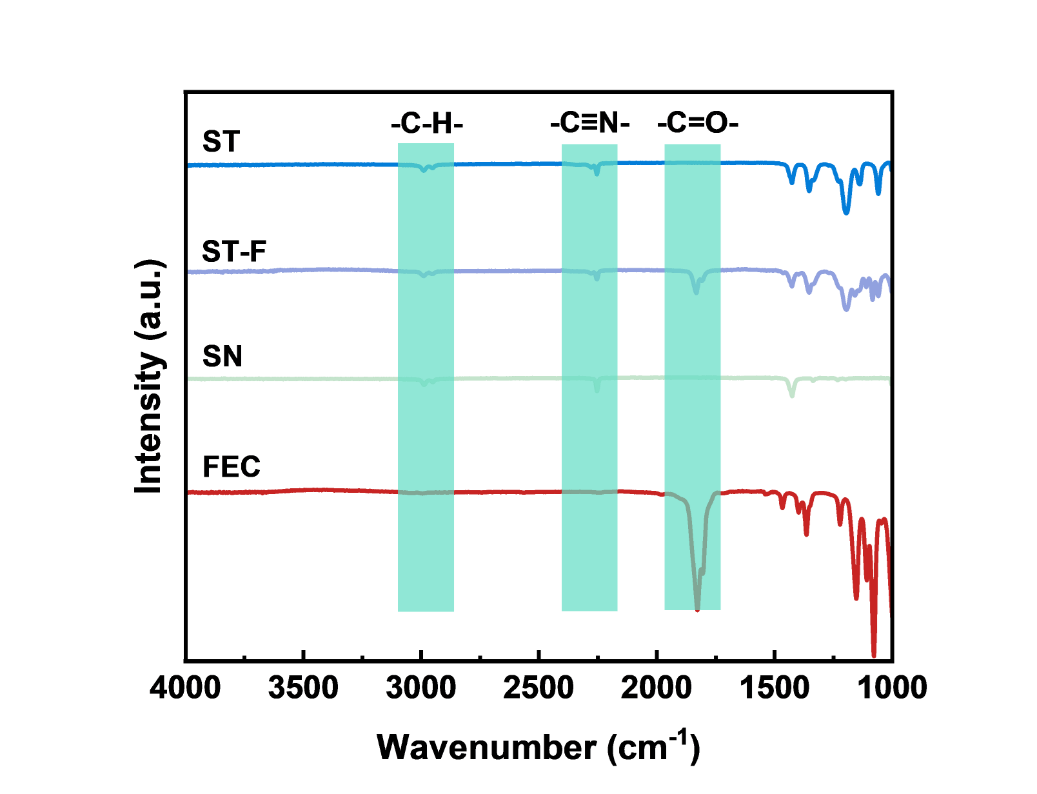


**Fig. S5** Fourier Transform Infrared Spectroscopy test


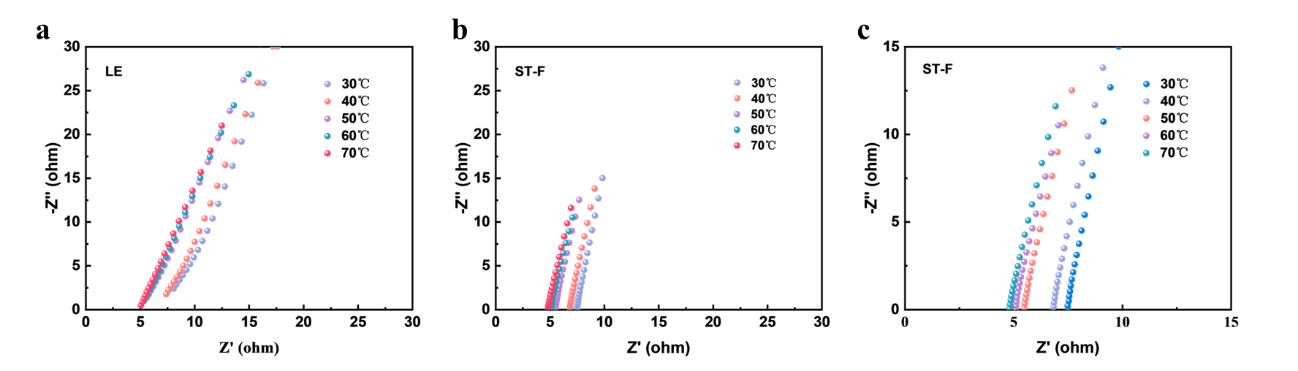


**Fig. S6** Variable temperature conductivity Test. (**a**) LE, (**b**) ST, (**c**) ST-F


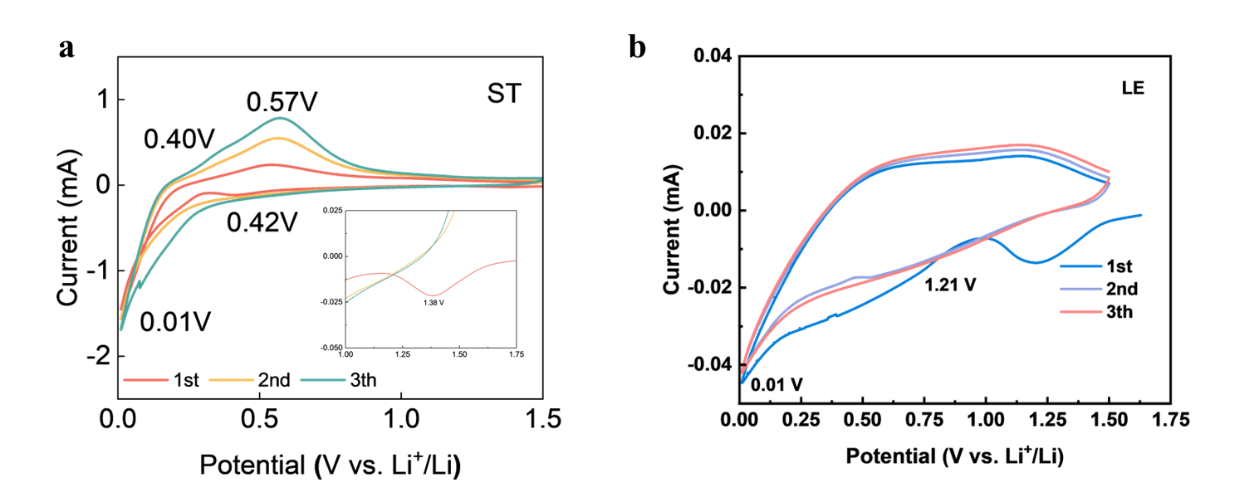


**Fig. S7** Variable temperature conductivity Test. (**a**) ST, (**b**) LE


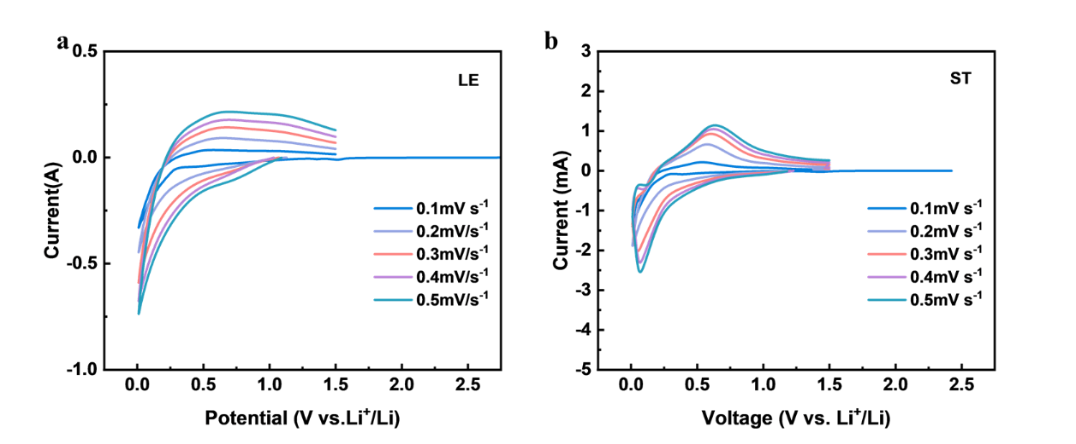


**Fig. S8** CV curves of SiO anode in ST-F electrolyte with different sweep speeds. (**a**) LE, (**b**) ST


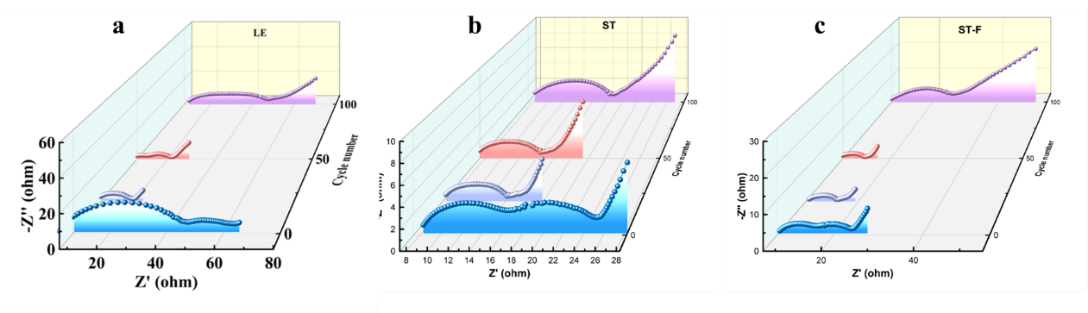


**Fig. S9** SiO anode impedance during cycling in electrolyte of 1st、20th、50th and 100th. (**a**) ST, (**b**) ST-F, (**c**) ST-F, (**d**) LE


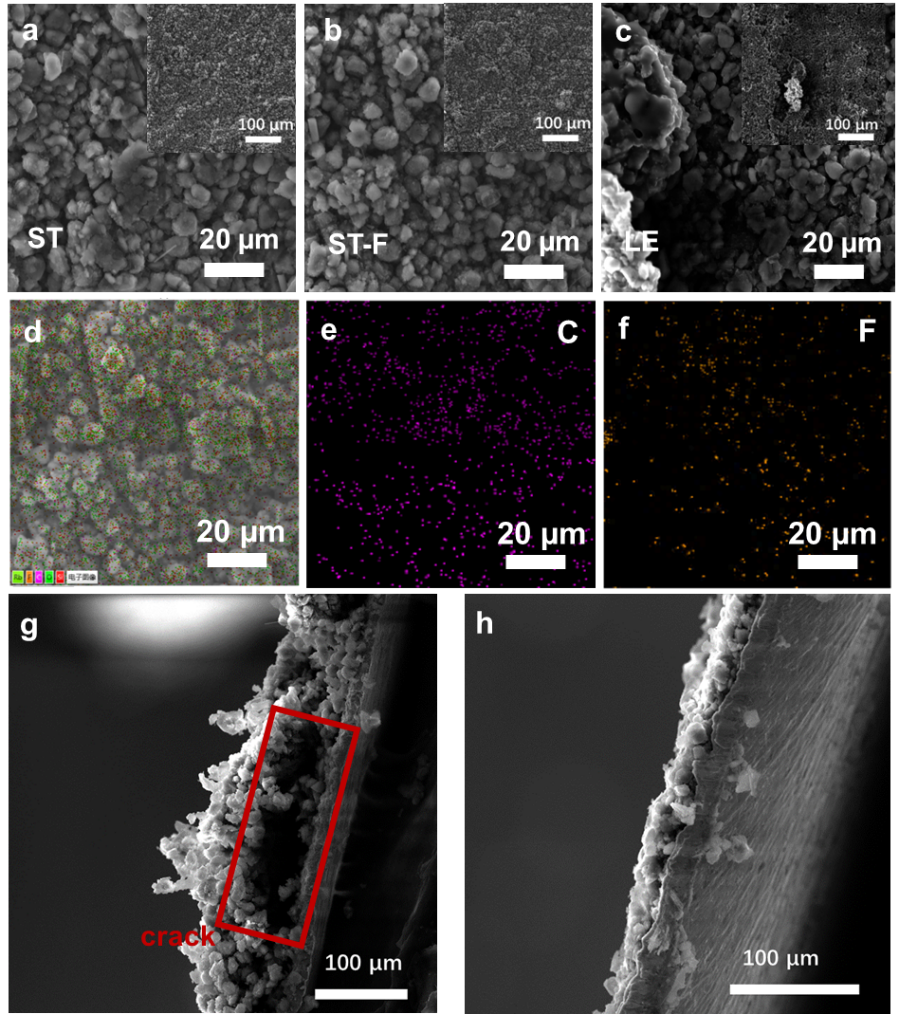


**Fig. S10** Surface morphology of SiO anode after cycling. (**a**) ST (**b**) ST-F, (**c**) LE. EDS energy spectrum analysis (**d**) EDS energy spectrum of ST- F, (**e**) C, (**f**) F. Cross-section after cycling. (**g**) ST, (**h**) ST-F


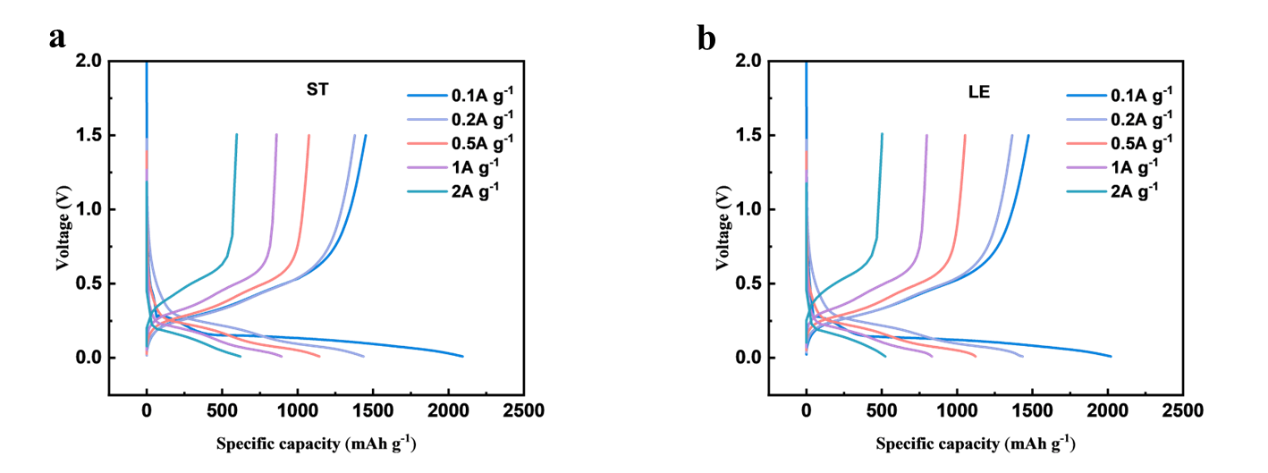


**Fig. S11** Charge/discharge curves at different current densities. (**a**) ST, (**b**) LE


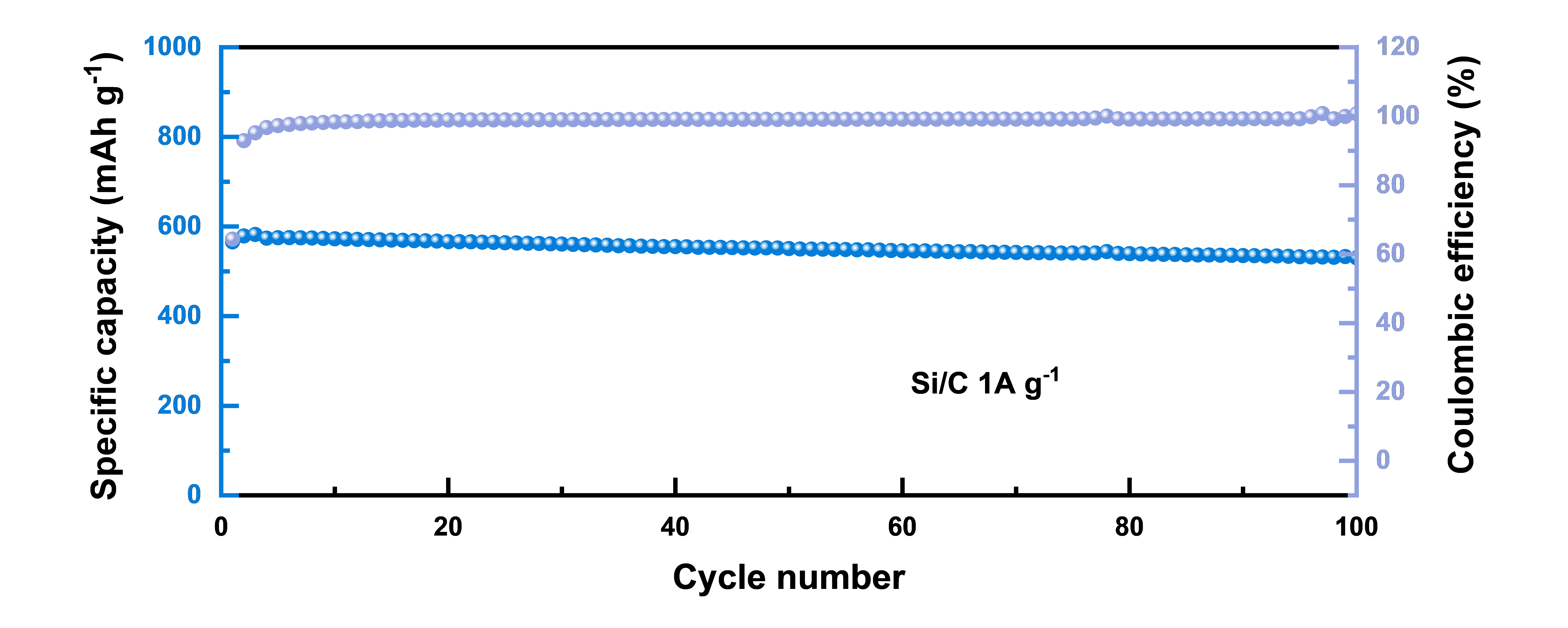


**Fig. S12** SiC cycling performance in ST-F electrolytes


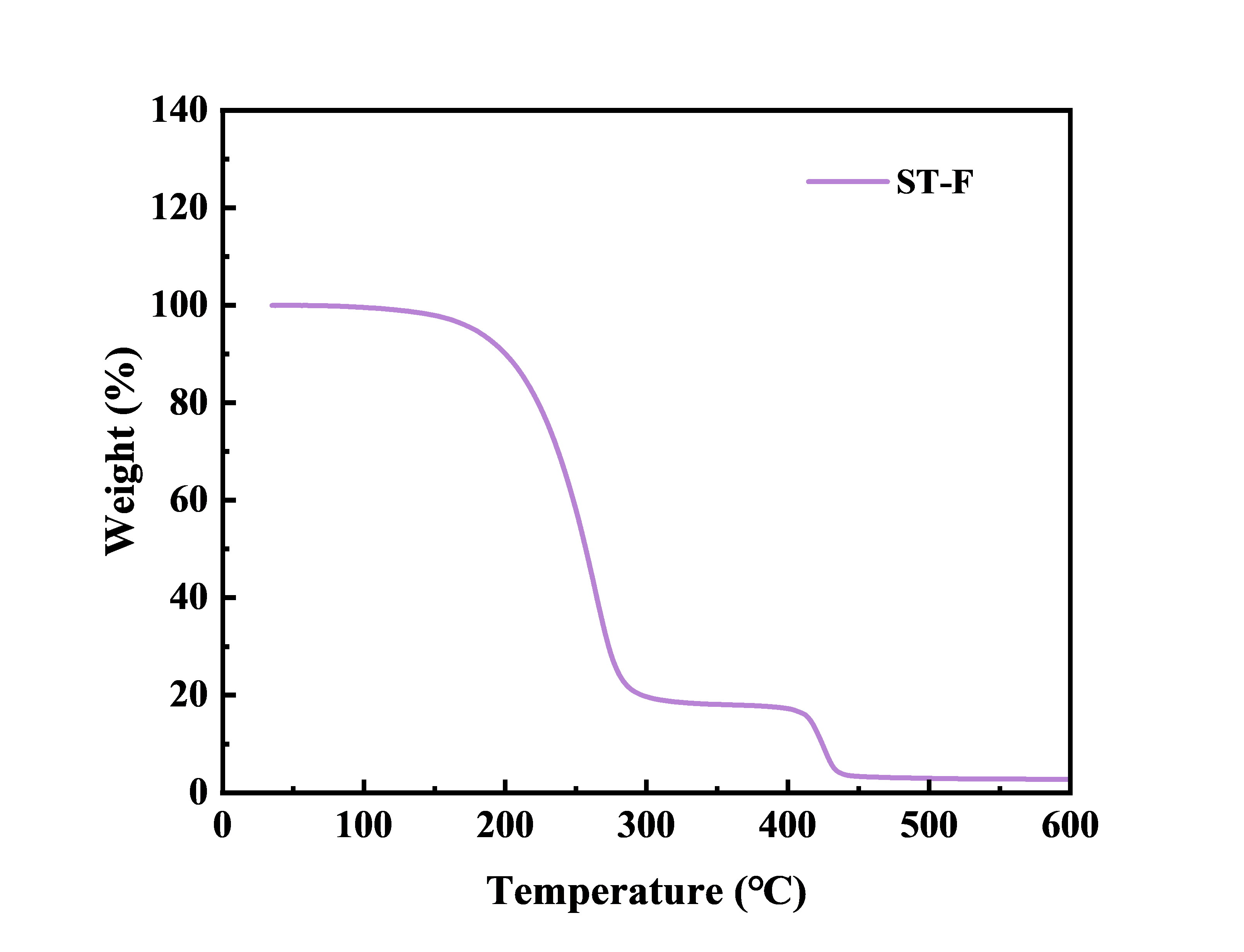


**Fig. S13** TG curve of ST-F electrolyte
